# Approximation to the phylogenetic position and morphological variability of Levanderina fissa in Acapulco Bay, with observations on diatom feeding behaviour

**DOI:** 10.1099/acmi.0.000904.v4

**Published:** 2026-05-11

**Authors:** Sinuhé Hernández Márquez, María Eugenia Zamudio-Resendiz, María Luisa Nuñez-Resendiz, Kurt Martin Dreckmann, Nelly María López Ortiz, Laura Margarita Márquez-Valdelamar, Abel Sentíes

**Affiliations:** 1 Doctorado en Ciencias Biológicas y de la Salud, Universidad Autónoma Metropolitana, Mexico, Mexico; 2 Laboratorio de Fitoplancton Marino y Salobre, Departamento de Hidrobiología, Universidad Autónoma Metropolitana, Iztapalapa, Mexico; 3 Laboratorio de Macroalgas Marinas y Salobres, Departamento de Hidrobiología, Universidad Autónoma Metropolitana, Iztapalapa, Mexico; 4 Laboratorio de Secuenciación Genómica, LaNaBio, Pabellón Nacional de la Biodiversidad, Instituto de Biología, Universidad Nacional Autónoma de México, Mexico, Mexico

**Keywords:** interactions, LSU, large subunit ribosomal RNA, mixotrophic, small subunit ribosomal RNA, speciation, SSU, TEM, Transmission Electron Microscopy

## Abstract

*Levanderina fissa*, a globally distributed mixotrophic dinoflagellate, is known for its ability to photosynthesize and phagocytize micro-organisms. This study aims to approximate the phylogenetic position and explore the morphological variability of *L. fissa* in Acapulco Bay, Mexico, while providing observations on its diatom feeding behaviour. Phylogenetic analysis based on 28S and 18S ribosomal DNA sequences suggests the existence of a monophyletic group within the Gymnodiniales, though with low phylogenetic resolution, indicating potential cryptic species within the genus. Morphological analysis reveals variability that may be influenced by regional environmental factors. Observations on diatom ingestion provide further, albeit limited, insights into the mixotrophic behaviour of *L. fissa*. These findings emphasize the need for further research to better understand the species’ ecological role and phylogenetic position, particularly concerning its feeding strategies in tropical ecosystems.

## Data Summary

The data are available in Table 1.

**Table 1.** Molecular sequences used in this study Sequences generated in this study are in bold, and others are from GenBank.

Species	Collection data (country: site; collector(s); date)	GenBank gene 28S access no.	Collection data (country: site; collector(s); date)	GenBank gene 18S access no.
	(–) Indicates no data		(–) Indicates no data	
*Akashiwo sanguinea*	–	ON287028	–	AY421771
*Baldinia anauniensis*	–	EF052683	–	EF052682
*Ceratoperidinium margalefii*	Spain: Catalonia; July 2011	KF245455	–	–
*Cochlodinium* cf *convolutum*	Spain: Catalonia; November 2012	KF245460	–	MF948385
*L. fissa*	–	AY036077	–	GU362426
*L. fissa*	–	EF192407	–	AY421786
*L. fissa*	–	EF192410	USA: North Carolina, New River Estuary	AY721981
*L. fissa*	China: Xiamen Harbor; 2004	DQ084521	Catalan coast; A. Rene	KP790163
*L. fissa*	USA	DQ847432	–	AF274263
*L. fissa*	–	DQ997780	China: Xiamen Harbor, Fujian Province; 2004	DQ084522
*Heterocapsa rotundata*	–	AF260400	–	–
*Karenia cristata*	South Africa	AY243963	–	–
*L. fissa*	–	EF613354	USA	DQ847433
***L. fissa***	**Mexico: Acapulco; S. Hernández-Márquez; June 2019**	**OR029321**	**Mexico: Acapulco; S. Hernández-Márquez; June 2019**	**PP152259**
***L. fissa***	**Mexico: Acapulco; S. Hernández-Márquez; June 2019**	**OR029322**	**Mexico: Acapulco; S. Hernández-Márquez; June 2019**	**PP152260**
***L. fissa***	**Mexico: Acapulco; S. Hernández-Márquez; June 2019**	**OR029323**	**Mexico: Acapulco; S. Hernández-Márquez; June 2019**	**PP152261**
***L. fissa***	**Mexico: Acapulco; S. Hernández-Márquez; June 2019**	**PP158603**	**Mexico: Acapulco; S. Hernández-Márquez; June 2019**	**PP152262**
***L. fissa***	**Mexico: Acapulco; S. Hernández-Márquez; June 2019**	**PP158604**	**Mexico: Acapulco; S. Hernández-Márquez; June 2019**	**PP152263**
*L. fissa*	France: Concarneau Bay; E. Nezan; June 2009	KJ508395	–	AY443015
*L. fissa*	Spain: Catalonia; A. Reñé	KP790223	–	OL439711
*L. fissa*	EEUU: New River; C. Tomas; January 2009	MW774165	–	EF492498
*Moestrupia oblonga*	Spain; G. Hansen; October 2004	JF272764	Japan: Okinawa, Itoman, Odo; 2013	LC025897
*Nusuttodinium poecilochroum*	Japan: Hokkaido; R. Onuma; May 2012	LC027065	Japan: Hokkaido, Hokuto; R. Onuma; 2012	LC027047
*S. natans*	Spain: Canary Islands; October 2004	EU315917	Japan: Kochi, Otsuki-cho	AB704015
*Warnowia* sp.	Canada: British Columbia	FJ947042	–	–
*Borghiella tenuissima*	–	AY571374	–	–

## Introduction

Dinoflagellates are a diverse group of unicellular micro-organisms prevalent in both marine and freshwater environments. While many species within this group are photosynthetic, many also have the additional capability to feed on various micro-organisms, a phenomenon known as mixotrophy [1,3]. This mode of nutrition has been previously documented in numerous dinoflagellate species, with diets ranging from bacteria and diatoms to ciliates and other dinoflagellate species [3,7].

*Levanderina fissa* (Levander) Moestrup, Hakanen, Gert Hansen, Daugbjerg and M. Ellegaard, a naked, mixotrophic dinoflagellate, exhibits a widespread distribution across both tropical and temperate environments globally [8,10]. It was first described by Levander in 1894 as *Gymnodinium fissum* Levander, within the order Gymnodiniales. Subsequently, [10] identified seven morphologically similar species as synonyms of *L. fissa*. This synonymy was based on morphological and molecular evidence, rendering *L. fissa* currently a monospecific genus with an uncertain status within Gymnodiniales.

Since the delineation of *L. fissa*, the first evidence of mixotrophy in this species was recorded by Levander [11], illustrated by a cell diagram depicting an ingested diatom. Subsequently, the certainty of its feeding behaviour and mixotrophy was affirmed through electron microscopy, revealing ingestion of unidentified organisms, in addition to the ciliate *Strombidium* Claparède and Lachmann and a small dinoflagellate from the genus *Gyrodinium* Kofoid and Swezy. Given the descriptive and historical evidence, *L. fissa* has been reported as an active predator of other organisms such as bacteria and protists, exhibiting non-selective feeding habits [6]. In a study by Lee *et al*. [12], it was demonstrated that *L. fissa* and other common dinoflagellates can feed on the ciliate *Mesodinium rubrum* (Lohmann). Furthermore, Liu *et al*. [13] analysed the impact of light and temperature on the growth and feeding of *L. fissa*.

This study focuses on approximating the phylogenetic position of *L. fissa* and examining its morphological variability within Acapulco Bay. Additionally, the study includes observations on the species' feeding behaviour, particularly its interaction with *Minutocellus polymorphus* (Hargraves and Guillard) Hasle, Stosch and Syvertsen. By analysing the phylogenetic relationships and morphological characteristics of *L. fissa* in this tropical region, we seek to enhance the understanding of this species’ variability and ecological role.

## Methods

### Study area and sampling

**Fig. 1.**
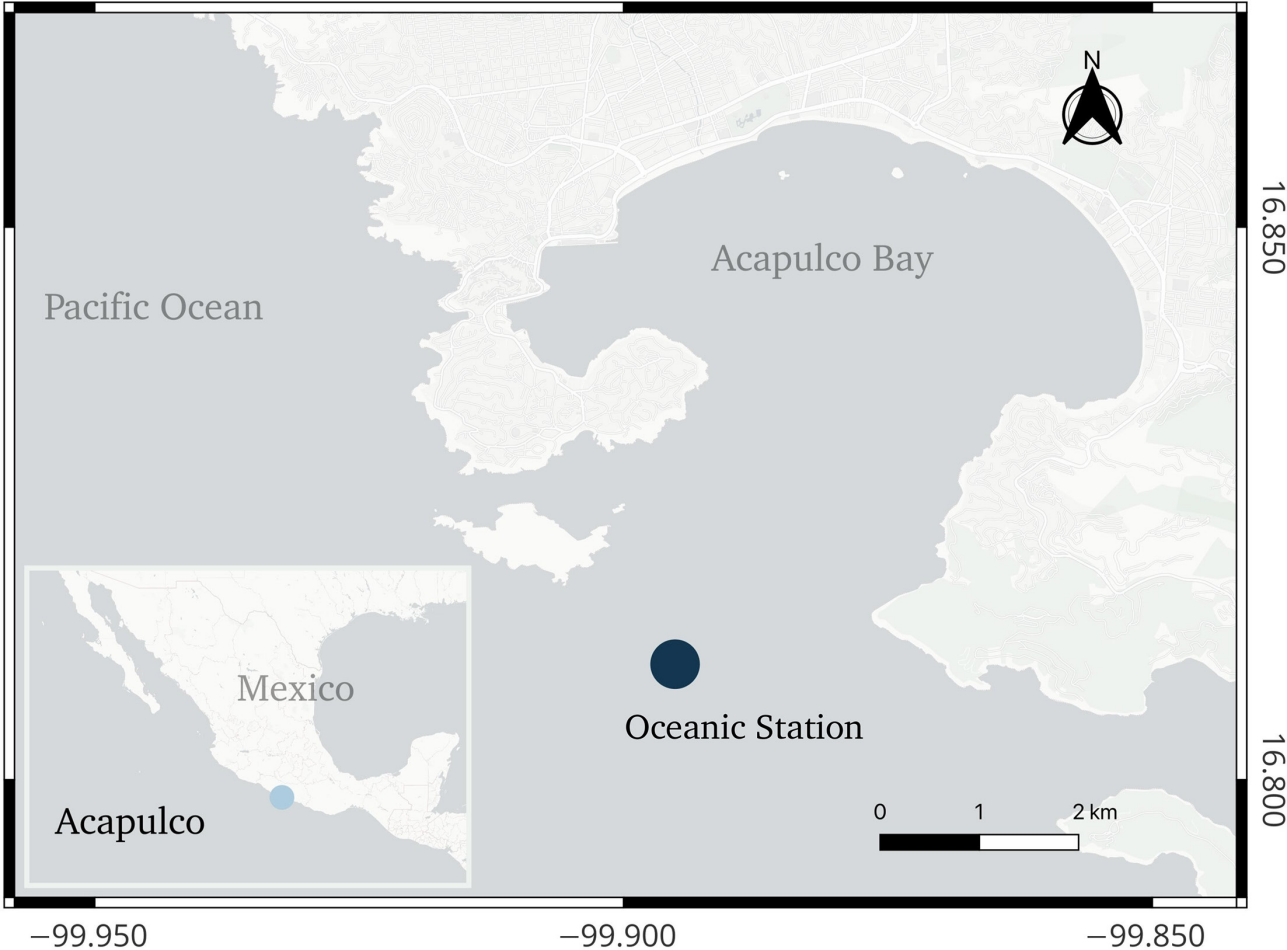
Map of Acapulco Bay showing the collection area (Oceanic Station).

Specimens of *L. fissa* were obtained from the oceanic waters off Acapulco, Guerrero, Mexico, during the rainy season on 8 June 2019 (Fig. 1). Sampling involved collecting surface water with a 3-l Van Dorn bottle, while additional samples were retrieved from depths of 3 and 5 m and transferred to a sterilized 10-l container. To prevent temperature fluctuations, the collected samples were immediately placed into insulated containers for later examination in the laboratory.

### Sample processing

Sample concentration was achieved through reverse filtration following the protocol outlined by Dodson and Thomas [14]. A 100 µl subsample was examined under a Leica DMS microscope (Germany) at 100× magnification, documenting the morphological characteristics of 24 cells representative of the sample’s diversity. Cells of interest were isolated using a finely calibrated micropipette, and monoclonal cultures were subsequently established. These cultures enabled detailed taxonomic assessment through ribosomal DNA sequencing and ultrastructural analysis [15,16].

### Cultivation conditions

The cells were cultivated in modified GSe medium (G medium enriched with selenium) following the protocol of Hernández-Márquez *et al*. [15], maintaining a 15 ml volume to replicate their native habitat. Environmental parameters were kept consistent, with salinity at 35 PSU, pH at 8.1 and a temperature of 29 °C. Light exposure was regulated at an intensity of 98 µmol m^−2^ s^−1^ under a 12:12 light/dark photoperiod.

### Single-cell PCR and sequencing

The identification of *L. fissa* was performed through phylogenetic analysis based on single-cell PCR techniques. The amplification of the 28S rRNA region employed the D1R-F forward primer (ACCCGCTGAATTTAAGCATA) and the D3B-R reverse primer (TCGGAGGGAACCAGCTACTA), following the method described by Scholin *et al*. [17] and Nunn *et al*. [18]. Simultaneously, the 18S rRNA region was targeted using the SR4 forward primer (AGGGCAAGTCTGGTGCCAG) and the SR9 reverse primer (AACTAAGAACGGCCATGCAC) as outlined by Matsuoka *et al*. [19]. Both amplification protocols followed the conditions specified by Hernández-Rosas *et al*. [16] with Maxima Hot Start PCR Master Mix.

The integrity of the PCR products was assessed through electrophoresis on 1% agarose gels, stained with GelRed® (Biotium) for enhanced visualization under UV light. Amplified fragments were purified with ExoSAP-IT (USB, Cleveland, Ohio, USA) to ensure high-quality sequencing. Bidirectional sequencing of both 28S and 18S rRNA regions was carried out using the same primers, with services provided by LaNaBio at UNAM.

### Phylogenetic analysis

The sequences obtained from the 28S and 18S rRNA genes were concatenated (Table 1). Sequence assembly and editing were completed using Sequencher v5.4.5 [20]. The final alignment incorporated sequences of *Levanderina* and related taxa from GenBank for both the 28S and 18S regions (Table 1) and was performed using BioEdit [21]. *Symbiodinium natans* Gert Hansen and Daugbjerg served as the outgroup. Phylogenetic reconstruction was carried out using both Bayesian Inference (BI) and maximum likelihood (ML) methods, applying codon partitioning. The K2+G model (Kimura’s two-parameter model with gamma distribution) was selected based on the likelihood ratio test using Topali V2 [22]. ML analysis employed RAxML [23] with branch support evaluated through 1,000 bootstrap replicates. BI analysis was performed in Mr. Bayes v3.2.2 [24] using four Markov chain Monte Carlo chains. Starting with a random tree, sampling occurred every 500 generations over 5 million generations, with stabilization reached after 1 million generations. A burn-in of 25% of the sampled trees was applied. Pairwise distance values (*P*-distances) were computed with mega V5 [25].

### Inoculation analysis

Following the establishment of monoclonal cultures, *M. polymorphus* (*n*=60) from the PhycoProt Herbarium strain C-047 (PHYCOP in Thiers [26]), characterized both morphologically and molecularly in Hernández-Márquez *et al*. [15], was inoculated with the *L. fissa* (*n*=1,784) strain. This co-culture was maintained for 4 days under standard conditions, followed by ultrastructural analysis.

### Cellular ultrastructure

Ultrastructural analyses were performed on 40 cultured cells isolated using a capillary-reduced micropipette. Cells were fixed at 4 °C with Karnovsky’s fixative [27], neutralized in 0.1 M sodium cacodylate for 1 h and post-fixed with 0.01% osmium tetroxide in Milli-Q water for 2 h. Following a 1 h rinse in distilled water, the cells were dehydrated in an ethanol gradient, based on the protocol in [15]. Samples were stained with toluidine blue, sectioned at 0.1 µm using a microtome and examined under a Jeol JSM7600-F Transmission Electron Microscope.

## Results

### Phylogenetic analysis

**Fig. 2.**
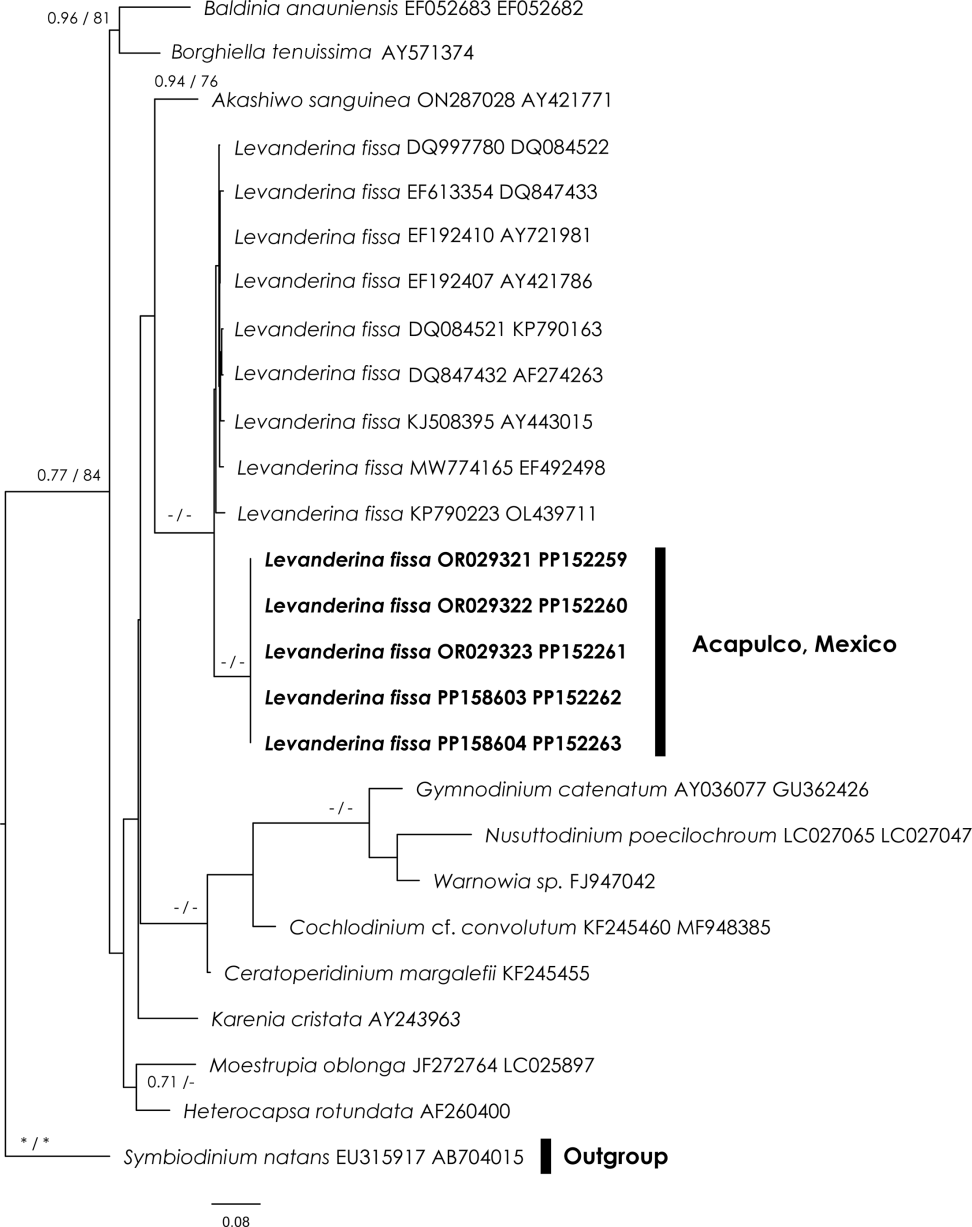
BI topology based on 28S and 18S sequence data. The values on the branches are BI values (left) and ML bootstrap. Asterisks indicate full support (ML=100%, BI=1.0%), and dashes indicate values below 70%. Sequences generated in this study are in bold. Scale: substitutions per site.

The concatenated sequence alignment, totalling 1,594 bp, produced ML and BI trees with consistent topologies (Fig. 2). These trees grouped the sequences of *Levanderina* obtained in this study with those from GenBank into a monophyletic clade. However, this clade did not receive strong phylogenetic support and is situated within the taxonomically uncertain order Gymnodiniales. Notably, genetic variation was observed within the *L. fissa* clade, with our samples forming a distinct genetic subgroup, supported by the highest phylogenetic confidence (Fig. 2). Although there was no genetic variation among our sequences, the genetic distance between them and other *Levanderina* sequences from GenBank varied between 1.3 and 2.4%.

### Morphological analysis

**Fig. 3.**
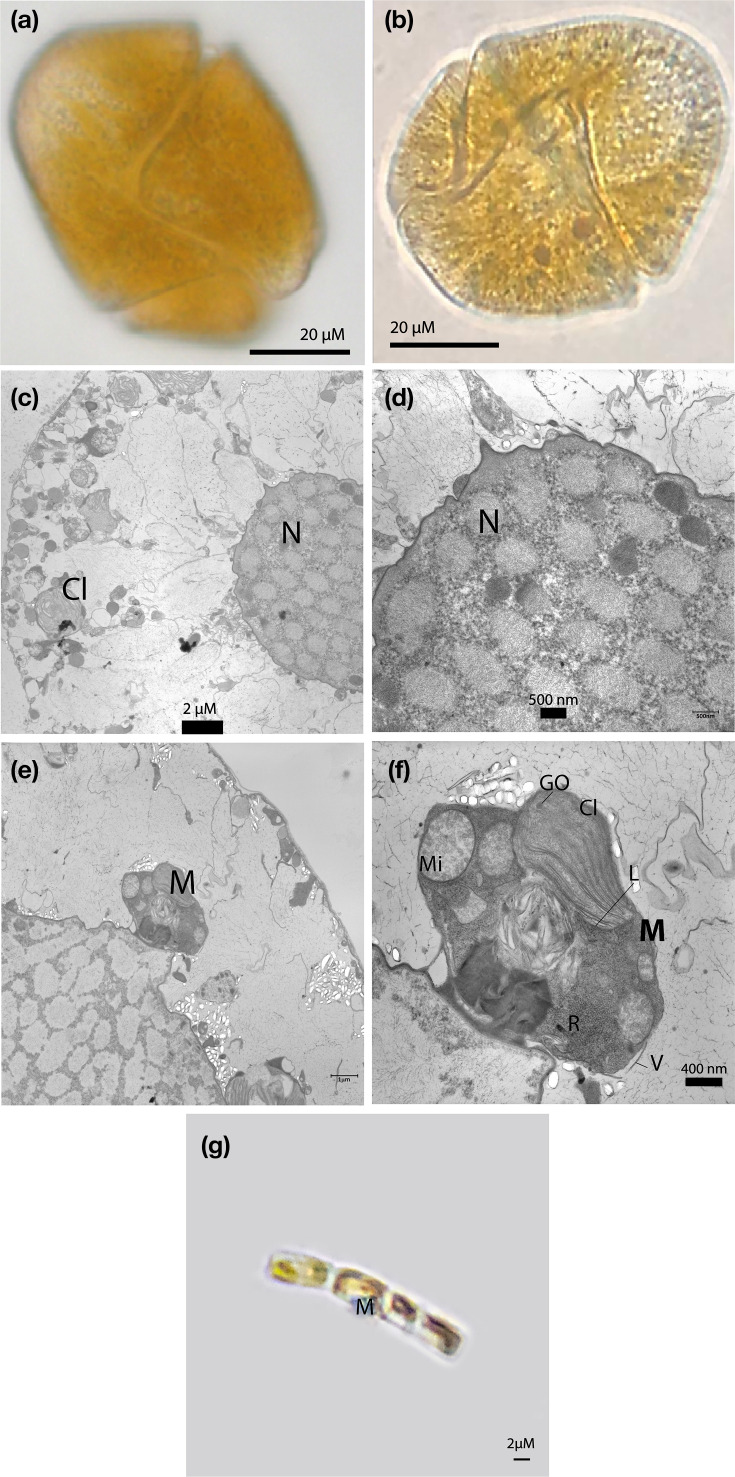
Scales are indicated in the image. (**a**) General view of the *L. fissa* cell obtained in culture. (**b**) General view of the *L. fissa* cell from a field sample. (**c**) Micrograph of the ultrastructure of *L. fissa*. (**d**) Close-up of the nucleus. (e) The nucleus is shown alongside the diatom. M*, M. polymorphus* ingested. (**f**) Close-up of the ultrastructure of *M. polymorphus*. (**g**) Photograph of the inoculated strain of *M. polymorphus* in bright field microscopy. The images display the N, nucleus; Cl, chloroplasts; Mi, mitochondria; R, ribosomes; V, valve; P, pyrenoid; GO, osmiophilic granules; L, lamella; and RP, periplasmic reticulum.

The morphological analysis was conducted using 74 cells, comprising 50 in culture and 24 in a natural environment. No significant differences were observed between the characterization in culture (Fig. 3a) and those from natural setting (Fig. 3b).

### *L. fissa* (Levander) Moestrup, Hakanen, G.Hansen, Daugbjerg and M.Ellegaard

In this study, the cells, devoid of any covering, measured 43–72 µm in length and 34–43 µm in width, with a trans diameter of 33.2–54.5 µm and a displacement of the girdle by ½. The parietal chloroplasts were 4.61–5.32 µm in length and 1.34–2.57 µm in width (Fig. 3c, d). The nucleus, containing ~60 chromosomes, measured 12.8–17.4 µm in length and 10.2–14.7 µm in width. The comparison with the Moestrup *et al*. [10] study is shown in Table 2.

**Table 2.** Comparison of the morphological characteristics of *L. fissa* between this study and Moestrup *et al*. [10]

*L. fissa*	This study	Moestrup *et al*. 2014
**Length**	43–73 µm	22.9–50 µm
**Width**	43 µm	16.5–34.8 µm
**Transdiameter**	33.2–54.5 µm	16.5–34.8 µm
**Arrangement of chloroplasts**	Parietal	Radiating from the centre of the cell
**Size chloroplasts**	4.61–5.32×1.34–2.57 µm	4.8×1.6 µm
**Size nucleus**	12.8–17.4×10.2–14.7 µm	6×5.7 µm
**Cingular displacement**	1/2, 13.74, 15.57	from 1/2 to 1/3
**Chromosomes**	≈60	≈60

### Co-culture analysis

In Fig. 3(e, f), the nanoplanctonic diatom *M. polymorphus* is visible on the side of the nucleus of *L. fissa*. At the lower zone, remnants of a digested valve are evident, causing cell deformation. Organelles are evenly distributed, including an open chloroplast (1.67 µm in length and 0.386–1.58 µm in width, considering the rupture of the chloroplast), multiple mitochondria (120–594 nm), a nucleus (0.5–1.7 µm) and the rough endoplasmic reticulum. Fig. 3(g) displays the strain of *M. polymorphus* that was inoculated.

## Discussion

### Molecular phylogeny and morphological variability

Our study emphasizes the importance of species characterization, particularly in the molecular and morphological identification of *L. fissa*. Phylogenetic analysis based on concatenated 18S and 28S rDNA sequences placed *L. fissa* within a monophyletic group in the Gymnodiniales, in agreement with previous studies on dinoflagellate phylogeny [10,28, 29]. However, the low resolution observed suggests the presence of cryptic species within the genus, highlighting the need for further investigation using additional genetic markers such as ITS and 5.8S rDNA. Notably, no significant genetic differences were found between *L. fissa* sequences from GenBank and those obtained in this study, suggesting a high degree of genetic uniformity within the species. Nonetheless, the conserved nature of the 28S and 18S genes, as noted in other dinoflagellate studies [28,30], poses challenges in resolving phylogenetic relationships without incorporating additional genomic regions.

Minor variations observed in the concatenated sequences may be attributed to geographic differences and the tropical climate, as well as the subtle morphological variations identified in our study. These findings underline the necessity of continued research to uncover genetic diversity within *Levanderina* and resolve the taxonomic status of this and other dinoflagellate groups. Moreover, our study corroborates the global pattern that morphology can vary according to geographic distribution, especially between temperate and tropical zones. The morphological distinctions observed between the *L. fissa* from Acapulco Bay and those described by Moestrup et al. [10] further suggest regional adaptations influenced by latitude, salinity and temperature. Such morphological variation, dependent on environmental factors, has been documented in cysts [31] and vegetative organisms, such as *Prorocentrum* Ehrenberg [32].

### Mixotrophy and diatom feeding behaviour

Regarding the diet of *L. fissa*, previous reports have documented its ability to phagocytize bacteria and protists, including ciliates and other dinoflagellates [5,7]. The mixotrophic nature of *L. fissa* was first described by Levander [11], with subsequent studies, such as Moestrup *et al*. [10], depicting diatom ingestion. However, until now, no study has reported or documented diatom inclusion in its diet. In our study, using cultures from Acapulco Bay, we successfully documented the ingestion of *M. polymorphus*, characterized extensively by Hernández-Márquez *et al*. [15]. While diatom ingestion has been reported in other mixotrophic dinoflagellates, such as *Oxyrrhis marina* Dujardin, *Karlodinium veneficum* (D.Ballantine) J.Larsen and *Margalefidinium polykrikoides* (Margalef) F.Gómez, Richlen and D.M.Anderson [6,33], it is infrequently mentioned in the literature. Unlike *Kryptoperidinium triquetrum* (Ehrenberg) Tillmann, Gottschling, Elbrächter, Kusber and Hoppenrath, which requires altered culture conditions for phagocytosis [7], *L. fissa* did not require such modifications to consume diatoms, indicating a potentially more adaptable feeding strategy.

Our study provides a novel finding by documenting the ability of *L. fissa* to phagocytize diatoms and efficiently digest their cellular contents and, potentially under certain conditions, to partially process components of their siliceous frustules, a phenomenon previously suggested for other dinoflagellates such as *Oxyrrhis marina* [34,35]. This capacity is particularly relevant, as it may contribute to the ecological success of mixotrophic dinoflagellates in nutrient-poor environments by enabling the exploitation of silica-containing prey and the potential release of silicic acid, as proposed for other organisms [36,37], despite the defensive role of the frustule against predation [38]. Conversely, from an alternative hypothesis, it may be considered unlikely that, in the cell illustrated in Fig. 3(f), the diatom frustule was completely dissolved while the diatom cytoplasm remained preserved; an equally plausible scenario is that the cytoplasmic contents of the diatom were digested, whereas the frustule, composed of highly resistant biogenic silica, initially persists as a remnant. Nevertheless, it cannot be excluded that, at later stages of the digestive process, partial weakening or dissolution of the frustule may occur. The absence of a clearly preserved food vacuole membrane in Fig. 3(f), probably associated with suboptimal fixation, limits a definitive interpretation of the micrograph. Consequently, additional ultrastructural observations using optimized fixation protocols and time-series experiments will be necessary to clarify whether *L. fissa* is capable of actively dissolving diatom frustules or whether its digestion is primarily restricted to the organic components of the prey.

### Future research

This study provides critical insights into the phylogenetic position and morphological variability of *L. fissa* in a tropical environment, along with novel observations on its feeding behaviour. While these findings significantly contribute to our understanding of this species, they also highlight the need for future research to explore the genetic diversity and ecological roles of *L. fissa*. The presence of *L. fissa* in Acapulco Bay, a major tourist and fishing hub, may play a key role in harmful algal blooms, as observed in other toxic mixotrophic dinoflagellate species in Mexico [8,39, 40]. However, limitations in the current study, including the inability to measure prey–predator assimilation rates and observe phagocytosis in real time, underscore the need for advanced studies to fully understand the ecological and evolutionary dynamics of *L. fissa* in various habitats.

## Abbreviations

BI: Bayesian inference
ML: maximum likelihood
